# Greater weekly exercise volume is associated with lower prevalence of metabolic comorbidities, psychiatric conditions, and exertional symptoms in youth athletes undergoing pre-participation screening: an observational study

**DOI:** 10.3389/fcvm.2026.1864938

**Published:** 2026-07-07

**Authors:** Grace Qiu, Seong Kyu Kim, Douglas Corsi, Alexander G. Hajduczok, Imran Masood, Daniel Underberg, Brian Osler, Drew Johnson, David Shipon

**Affiliations:** 1 Department of Medicine, Perelman School of Medicine at the University of Pennsylvania, Philadelphia, PA, United States; 2 Sidney Kimmel Medical College, Thomas Jefferson University, Philadelphia, PA, United States; 3 Rutgers Robert Wood Johnson University Hospital, New Brunswick, NJ, United States; 4 Oklahoma Heart Institute, Tulsa, OK, United States; 5 Keck School of Medicine, Children’s Hospital of Los Angeles, Heart Institute, University of Southern California, Los Angeles, CA, United States; 6 Division of Cardiology, Jefferson Heart Institute, Thomas Jefferson University Hospital, Philadelphia, PA, United States

**Keywords:** exercise volume, exertional symptoms, pre-participation screening, psychiatric comorbidities, youth athletes

## Abstract

**Introduction:**

Physical activity has been associated with improved cardiovascular health outcomes in adults. However, the specific associations between physical activity volume and cardiovascular outcomes in youth athletes are less defined.

**Methods:**

This study analyzed data from the HeartBytes National Youth Database, compiled from Simon's Heart. Simon's Heart is a nonprofit organization that includes information from sports pre-participation examinations for adolescents. The dataset encompasses demographics, electrocardiogram (ECG) findings, and exercise-related symptoms. Youth athletes' self-reported weekly physical activity volume was classified into four levels: <2 h, 2–5 h, 5–10 h, and >10 h, with less than 2 h taken as the reference group. Given the physical diMerences among athletes aged 12 to 20, the sample was stratified into middle and high school athlete groups. Logistic regression analysis was performed to evaluate the association between weekly exercise volume and cardiovascular risk factors, controlling for the confounding eMects of age, race, sex, body mass index, and area-level socioeconomic status.

**Results:**

Among 7,048 youth athletes (median age 15.1 years; interquartile range: 13.6–16.5), most were male (60.7%) and White (85.5%). Increased weekly exercise volume was associated with lower odds of attention deficit hyperactivity disorder (ADHD), anxiety or depression, and obesity in both middle and high school athletes. Greater exercise volume high school athletes, though these did not reach statistical significance. Greater weekly physical activity volume was associated with lower prevalence of cardiovascular disease risk factors, psychiatric comorbidities, and exertional symptoms in youth athletes. Given the cross-sectional design, causal relationships cannot be established and further studies are needed to clarify the clinical significance of these findings.

## Introduction

Despite health improvements at the global level, the burden of cardiovascular disease (CVD) has remained constant and is the leading cause of death worldwide (1). While clinical manifestations of CVD tend to present in adulthood, earlier detection of cardiovascular manifestations in childhood and adolescence has increased (2). Therefore, it remains important to establish a better understanding of the heart in children and adolescents.

The impact of exercise on the adult heart has been well studied and has been proven to be associated with decreased risk of cardiovascular disease and death (3). The adaptations of the heart can depend on the type, volume, and intensity of exercise, as well as the years of exercise (4). However, the physiological and functional adaptations of the child athlete's heart are not well understood (5). Although some structural and electrical changes similar to those of adult athlete hearts have been observed in child athlete hearts (6–11), there is no definitive evidence to delineate the difference between exercise-induced remodeling and pathological changes of the heart. Notably, in adults, the relationship between exercise volume and cardiac morbidity has been described as U-shaped, with both sedentary behavior and extreme exercise volumes associated with adverse cardiovascular outcomes (12). Whether a similar relationship exists in young athletes remains unknown, further highlighting the need to better understand the associations between exercise volume and cardiovascular health in children and adolescents.

This study explores the association between exercise volume measured by weekly physical activity hours and the cardiovascular and non-cardiovascular risk factors, symptoms, and comorbidities of youth athletes. The study also assessed exercise-induced cardiac changes detected by electrocardiogram (ECG).

## Materials and methods

### Study design

This was a cross-sectional observational study that analyzed data from middle school (aged 12 to <14 years) and high school (aged 14–20 years) youth athletes. Age groups were defined based on typical school classification used in pre-participation physical exam (PPE) screening programs rather than physiologic pubertal age. For this study, “youth athlete” refers to any adolescent presenting for a PPE, regardless of weekly activity level. This includes individuals across a spectrum of activity levels, ranging from minimal weekly exercise to highly active athletes, reflecting a heterogeneous population that undergoes routine PPE screening. The study aimed to investigate associations between weekly physical activity volume and cardiovascular health metrics.

### Data source

This study involving human participants was reviewed and approved by the Institutional Review Board of Thomas Jefferson University (Protocol No. 20E.1254, approved December 23, 2020) under exempt review with waiver of authorization. The studies were conducted in accordance with local legislation and institutional requirements. Written informed consent for participation was provided by participants' legal guardians or next of kin. No animal studies are presented in this manuscript and no potentially identifiable data are presented in this study.

Data were obtained from the HeartBytes National Youth Database from Simon's Heart, a nonprofit organization dedicated to reducing sudden cardiac death (SCD) in young individuals. The dataset contains information from pre-participation screening events held across multiple locations between August 2014 and July 2021. Adolescents voluntarily attended these screening events, which were organized as community-based cardiac screening opportunities for youth seeking clearance for sports participation. Participants underwent cardiac evaluations as part of the standard PPE process.

### Participant data collection

Participants self-reported demographic and medical history details, including age, sex, race, and residential address, along with self-reported information about weekly physical activity volume and any exercise-related symptoms. Data entry was standardized to ensure accuracy and consistency across multiple screening sites.

### Measurement data

During the screenings, clinical evaluations were performed, including measurements of height, weight, and blood pressure. A detailed physical examination was conducted, with auscultation to detect heart murmurs. Electrocardiograms (ECGs) were also performed and interpreted by on-site cardiologists using established guidelines, transitioning from the Seattle Criteria before 2017 to the International Criteria for ECG interpretation in athletes thereafter (13). While the term “ECG abnormality” colloquially refers to ECG findings that deviate from the norm, many of these findings are physiologic in athletes.

### Independent variables

The primary independent variable of interest was weekly physical activity volume, defined as self-reported cumulative numbers of hours per week (from Monday to Sunday) spent engaging in physical activity within the past year. Participants were categorized into four groups based on this volume: <2 h, 2–5 h, 5–10 h, and >10 h per week. This measure reflects activity volume and not exercise intensity. Parameters like exercise effort level or metabolic equivalents were not collected. These exercise volume categories were pre-defined by the HeartBytes screening questionnaire and could not be modified for the analysis.

### Confounding variables

Potential confounding variables include age, race, sex, BMI, and socioeconomic status (SES). To account for SES, we utilized the Distressed Communities Index (DCI), an area-based metric to categorize community economic well-being into five quintile-based tiers: Prosperous, Comfortable, Mid-tier, At Risk, and Distressed (14). Each participant's residential zip code was mapped to its corresponding DCI tier to approximate for socioeconomic environment. These variables were chosen as confounders to control for based on their established associations with both physical activity levels and cardiovascular outcomes in youth populations. Pubertal stage, sport type classification, competitive level, and individual-level socioeconomic data were not collected as part of the HeartBytes screening protocol and could not be included as covariates. Family history of cardiovascular conditions was collected but had a high proportion of missing responses and was therefore excluded from the primary analysis.

### Outcome variables

The main outcomes include various comorbidities, exercise-induced symptoms, physical exam findings, and ECG abnormalities. The comorbidities of interest include asthma, elevated blood pressure, hypercholesterolemia, attention deficit hyperactivity disorder (ADHD), anxiety or depression, anemia, and obesity. Hypercholesterolemia was based on prior diagnosis recall and was not derived from lipid measurements obtained during the screening. Obesity was defined as a BMI at or above the 95th percentile for age and sex, calculated directly from individually measured height and weight. All comorbidities with the exception of obesity were recorded based on participant or guardian recall of prior clinician diagnoses and reported as binary yes/no variables. The exercise-induced symptoms include easily fatigued, chest pain, dyspnea, syncope, and palpitations. Exercise-induced symptoms were self-reported on the PPE questionnaire and reflect symptoms experienced within the past year. Physical examination findings, including murmurs, were assessed during the pre-participation screening. To address potential outcome overlap, participants with a reported asthma diagnosis were excluded from the dyspnea outcome analysis, as dyspnea may represent a symptom of asthma rather than an independent finding.

### Missing data and No event cases

In terms of covariates, participants with missing data for exercise volume, race, sex, BMI, or SES were excluded from the initial sample. The remaining participants with complete data formed the analytical database, which was divided into middle school and high school strata. Missing data within the final analytical database were restricted solely to outcome variables, specifically ECG and murmur outcomes in a small number of middle school and high school participants. Missing outcomes were handled on a per-outcome basis, whereby participants with missing values in a given outcome variable were excluded from that specific model only, ensuring consistency between descriptive and inferential analyses. All other outcomes had complete data. Exposure groups with no observed events for a given outcome were excluded from the analysis.

### Statistical analysis

Descriptive statistics were used to summarize demographic and clinical variables. Mean and standard deviation were used to summarize continuous, normally distributed variables. Non-normally distributed variables were reported with median and interquartile range (IQR). Categorical variables were presented as counts and proportions. Given the physical differences among athletes aged 12–20 years, the sample was stratified into middle school and high school groups, with separate logistic regression models fitted for each stratum per outcome. Logistic regression was performed using the glm() function in R with maximum likelihood estimation, adjusting for age, sex, race, BMI, and SES proxied by DCI. Age was included as a continuous variable calculated from date of birth in both strata to capture within-stratum developmental differences. The group of athletes who self-reported exercising less than 2 h per week was used as the reference group.

While the HeartBytes database contains a comprehensive range of ECG findings interpreted by on-site cardiologists, the prevalence of these findings was insufficient for meaningful multivariable analysis in this cohort. Accordingly, only T-wave inversion and ECG early repolarization had adequate event counts and were included as outcome variables for ECG analysis.

To control the false discovery rate (FDR) during multiple hypothesis testing, raw *p*-values were adjusted using the Benjamini-Hochberg method. Associations were considered statistically significant at an adjusted *p*-value threshold of 0.05 and a 95% confidence interval (CI). Tables 1, 2 summarize all statistical findings. For clarity, FDR-adjusted *p*-values are denoted as *p* unless otherwise specified.

**Table 1:** Multivariable logistic regression findings in middle school athletes.

Middle School Athletes (*n* = 2,168) | Reference: <2 h/week | Adjusted for age, sex, race, BMI, DCI				
Variable/Exposure	OR	95% CI	*p*-value (raw)	*p*-value adjusted (FDR)
Asthma				
2–5 h/week	0.960	0.514–1.897	0.901	0.937
5–10 h/week	1.075	0.594–2.075	0.820	0.874
>10 h/week	1.242	0.680–2.419	0.500	0.688
ADHD				
2–5 h/week	0.675	0.326–1.513	0.311	0.480
–10 h/week	0.396	0.196–0.872	0.014	0.052
>10 h/week	0.298	0.141–0.674	2.15 × 10^−3^	**0**.**010**
Anxiety or Depression				
2–5 h/week	0.852	0.376–2.192	0.717	0.816
5–10 h/week	0.433	0.193–1.106	0.056	0.123
>10 h/week	0.277	0.114–0.749	6.85 × 10^−3^	**0**.**029**
Hypercholesterolemia				
2–5 h/week	0.422	0.128–1.654	0.176	0.322
5–10 h/week	0.310	0.101–1.173	0.055	0.123
>10 h/week	0.191	0.050–0.810	0.017	0.056
Obesity				
2–5 h/week	0.470	0.261–0.875	0.014	0.052
5–10 h/week	0.245	0.138–0.449	2.52 × 10^−6^	**2.15** **×** **10^−5^**
>10 h/week	0.183	0.099–0.347	1.02 × 10^−7^	**1.57** **×** **10^−6^**
Chest Pain				
2–5 h/week	1.892	0.643–8.103	0.308	0.480
5–10 h/week	1.284	0.447–5.436	0.684	0.798
>10 h/week	1.029	0.346–4.431	0.964	0.964
Dyspnea				
2–5 h/week	0.608	0.286–1.391	0.213	0.373
5–10 h/week	0.414	0.200–0.928	0.023	0.065
>10 h/week	0.262	0.116–0.623	1.59 × 10^−3^	**8.15** **×** **10^−3^**
Easily Fatigued				
2–5 h/week	0.645	0.362–1.181	0.145	0.272
5–10 h/week	0.263	0.148–0.480	8.13 × 10^−6^	**6.26** **×** **10^−5^**
>10 h/week	0.197	0.105–0.375	5.13 × 10^−7^	**4.94** **×** **10^−6^**
Palpitations				
2–5 h/week	1.139	0.417–4.010	0.817	0.874
5–10 h/week	0.672	0.252–2.337	0.474	0.663
>10 h/week	0.712	0.261–2.512	0.548	0.736
Murmur				
2–5 h/week	1.410	0.370–9.268	0.660	0.782
5–10 h/week	2.082	0.606–13.122	0.325	0.491
>10 h/week	1.046	0.280–6.832	0.954	0.964
ECG: T-wave Inversion				
5–10 h/week	0.402	0.091–2.936	0.282	0.461
>10 h/week	0.054	0.002–0.643	0.023	0.065

Bold *p*-values indicate statistical significance at *α*, 0.05. OR, odds ratio; CI, confidence interval; DCI, Distressed Communities Index; FDR, false discovery rate; ADHD, attention deficit hyperactivity disorder; ECG, electrocardiogram. ECG T-wave inversion: the 2–5 h/week group is excluded because of no events; exposure rows show results for 5–10 and >10 h/week only.

**Table 2:** Multivariable logistic regression findings in high school athletes.

High School Athletes (*n* = 4,880) | Reference: <2 h/week | Adjusted for age, sex, race, BMI, DCI				
Variable/Exposure	OR	95% CI	*p*-value (raw)	*p*-value adjusted (FDR)
Asthma				
2–5 h/week	0.805	0.567–1.155	0.230	0.394
5–10 h/week	0.774	0.556–1.092	0.135	0.260
>10 h/week	0.948	0.688–1.328	0.752	0.839
Anemia				
2–5 h/week	0.742	0.229–2.827	0.631	0.782
5–10 h/week	0.951	0.339–3.389	0.930	0.955
>10 h/week	0.777	0.278–2.766	0.658	0.782
High Blood Pressure				
2–5 h/week	0.589	0.092–4.712	0.576	0.752
5–10 h/week	0.468	0.083–3.647	0.408	0.592
>10 h/week	0.332	0.058–2.630	0.234	0.392
ADHD				
2–5 h/week	0.600	0.398–0.918	0.016	0.056
5–10 h/week	0.421	0.284–0.634	2.29 × 10^−5^	**1.60** **×** **10^−4^**
>10 h/week	0.288	0.195–0.435	1.14 × 10^−9^	**2.19** **×** **10^−8^**
Anxiety or Depression				
2–5 h/week	0.710	0.467–1.096	0.114	0.225
5–10 h/week	0.318	0.207–0.495	2.39 × 10^−7^	**3.07** **×** **10^−6^**
>10 h/week	0.208	0.134–0.326	3.07 × 10^−12^	**7.89** **×** **10^−11^**
Hypercholesterolemia				
2–5 h/week	1.106	0.480–2.884	0.823	0.874
5–10 h/week	0.767	0.336–1.993	0.554	0.736
>10 h/week	0.364	0.150–0.983	0.033	0.084
Obesity				
2–5 h/week	0.694	0.449–1.096	0.108	0.218
5–10 h/week	0.604	0.400–0.933	0.019	0.061
>10 h/week	0.496	0.331–0.763	9.85 × 10^−4^	**5.42** **×** **10^−3^**
Chest Pain				
2–5 h/week	1.140	0.669–2.034	0.643	0.782
5–10 h/week	0.751	0.445–1.329	0.301	0.480
>10 h/week	0.581	0.345–1.028	0.050	0.116
Syncope				
2–5 h/week	1.425	0.444–6.327	0.588	0.755
5–10 h/week	1.339	0.444–5.793	0.644	0.782
>10 h/week	1.593	0.550–6.767	0.452	0.644
Dyspnea				
2–5 h/week	0.649	0.441–1.045	0.069	0.143
5–10 h/week	0.397	0.252–0.636	8.54 × 10^−5^	**5.06** **×** **10^−4^**
>10 h/week	0.302	0.192–0.486	4.08 × 10^−7^	**4.49** **×** **10^−6^**
Easily Fatigued				
2–5 h/week	0.461	0.316–0.679	7.37 × 10^−5^	**4.73** **×** **10^−4^**
5–10 h/week	0.242	0.165–0.357	5.03 × 10^−13^	**1.94** **×** **10^−11^**
>10 h/week	0.131	0.087–0.197	7.39 × 10^−23^	**5.69** **×** **10^−21^**
Palpitations				
2–5 h/week	1.055	0.657–1.752	0.829	0.874
5–10 h/week	0.512	0.316–0.854	7.98 × 10^−3^	**0**.**032**
>10 h/week	0.496	0.311–0.819	4.40 × 10^−3^	**0**.**020**
Murmur				
2–5 h/week	3.445	1.214–14.465	0.043	0.106
5–10 h/week	3.261	1.179–13.525	0.049	0.116
>10 h/week	3.843	1.411–15.826	0.024	0.065
ECG: T-wave Inversion				
2–5 h/week	1.499	0.216–29.720	0.720	0.816
5–10 h/week	2.704	0.515–49.945	0.345	0.511
>10 h/week	3.650	0.731–66.519	0.213	0.373
ECG: Early Repolarization				
2–5 h/week	0.171	0.022–1.065	0.058	0.123
5–10 h/week	0.175	0.036–0.935	0.028	0.075
>10 h/week	0.180	0.044–0.914	0.022	0.065

Bold *p*-values indicate statistical significance at *α*, 0.05. OR, odds ratio; CI, confidence interval; DCI, Distr*e*ssed Communities Index; FDR, false discovery rate; ADHD, attention deficit hyperactivity disorder; ECG, electrocardiogram.

As a sensitivity analysis, the interaction between school level and exercise volume was tested in a combined unstratified model. Age stratification was maintained based on the physiological differences between middle and high school athletes related to pubertal development and cardiac maturation (15).

## Results

**Figure 1:**
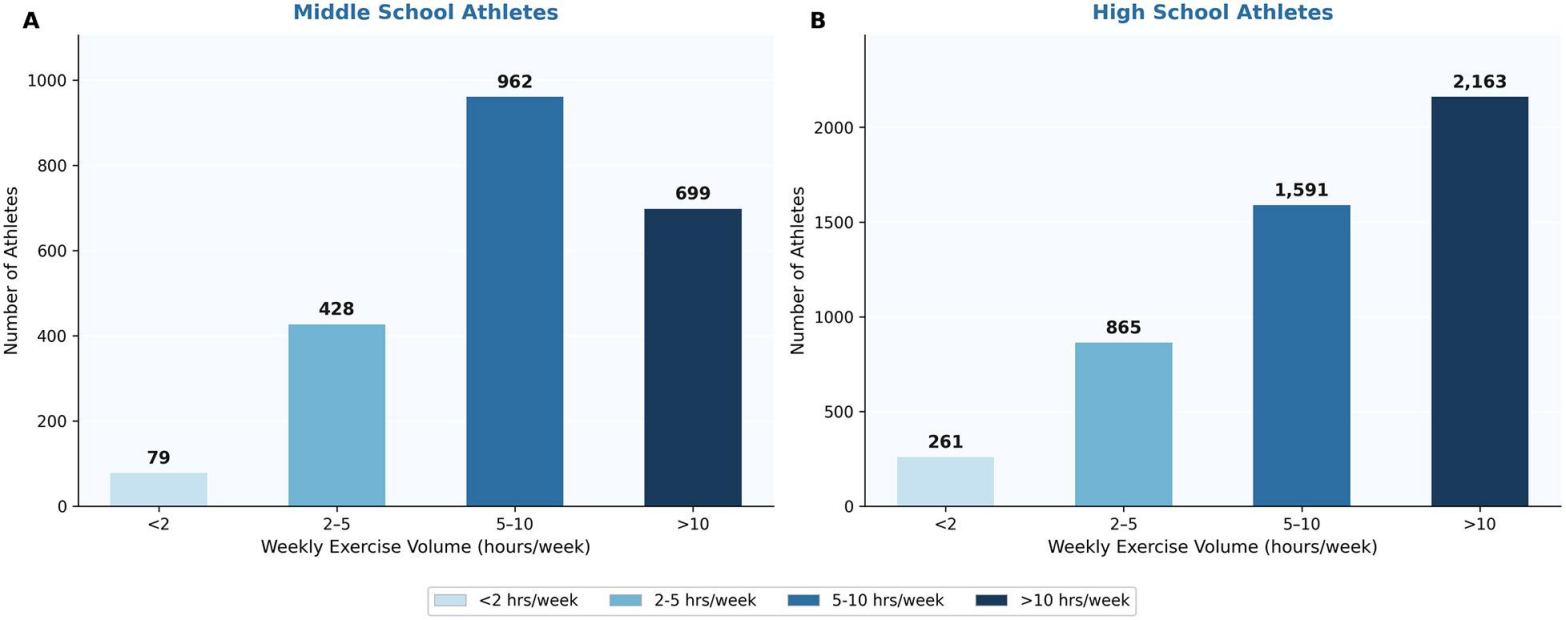
Distribution of athletes' exercise volume in **(A)** middle school and **(B)** high school.

Out of the 7,425 youth athletes included in the dataset, 7,048 individuals had complete demographic and covariate data, comprising 2,168 middle school athletes and 4,880 high school athletes. The majority of these athletes were White (85.5%) and male (60.7%), with 7.4% Black, 3.2% Hispanic/Latino, and 4.0% Asian/Pacific Islander participants. The age of the athletes ranged between 12 and 20 years, with a median age of 15.1 years (IQR: 13.6–16.5). The mean BMI was 21.6 kg/m^2^, with a standard deviation of 4.2 kg/m^2^. The majority of participants were from Pennsylvania, New Jersey, and Georgia. The distribution of exercise volume among athletes is shown in Figure 1.

**Figure 2:**
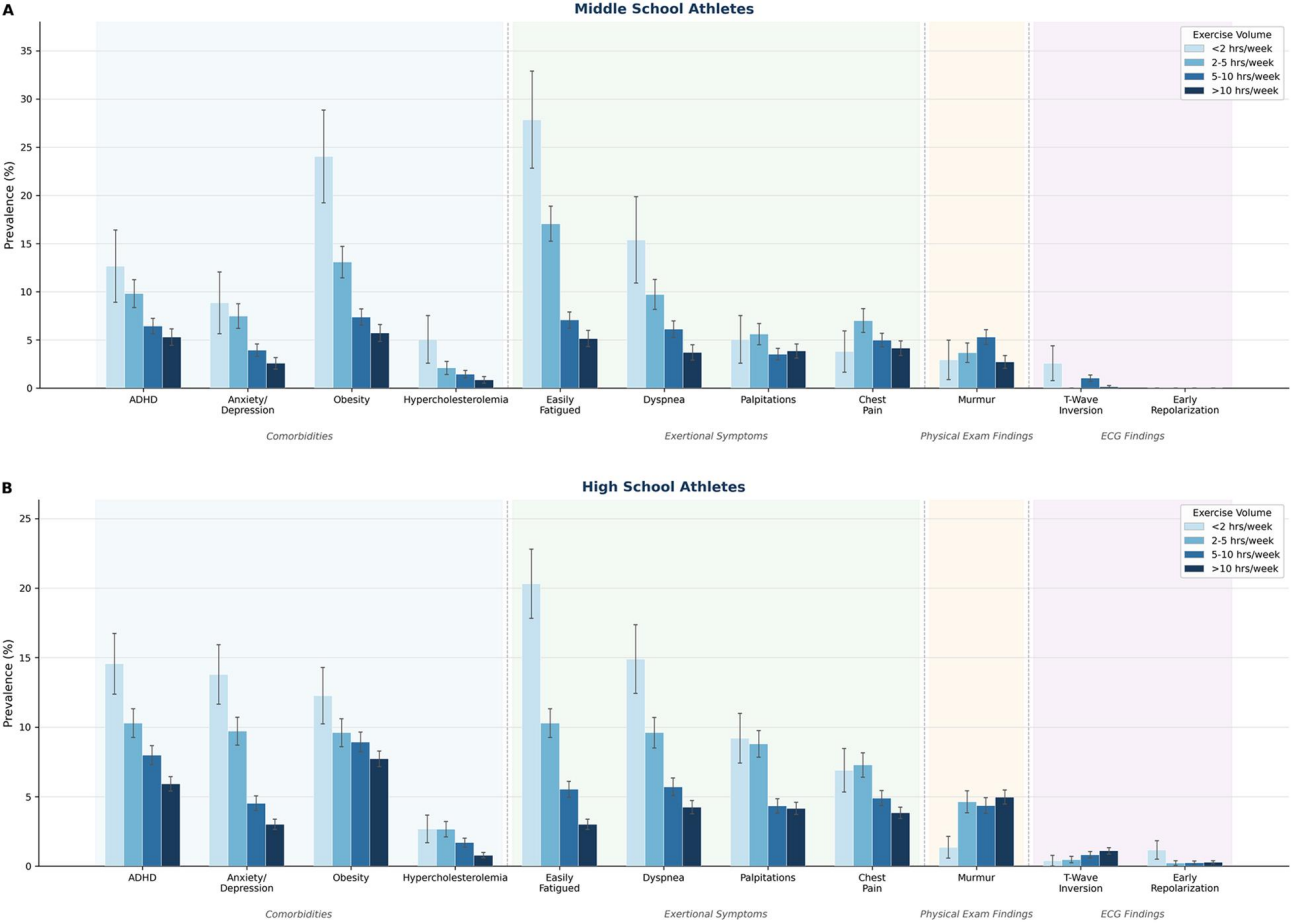
Prevalences of comorbidities, exertional symptoms, and ECG findings in middle school **(A)** and high school **(B)** athletes. Error bars represent standard error.

The prevalences of major comorbidities, exertional symptoms, and ECG findings among middle school and high school athletes are shown in Figure 2. Descriptive analysis based on Figure 2 showed that higher volume of weekly exercise was inversely associated with feeling easily tired and dyspnea on exertion in both middle school and high school athletes. Increasing weekly exercise volume from less than 2 h to over 10 h was found to be inversely associated with obesity in both middle school and high school athletes. A similar inverse association trend was observed for psychiatric comorbidities, including ADHD, anxiety, and depression. A higher prevalence of ECG early repolarization finding was found in high school athletes exercising less than 2 h per week.

### Logistic regression findings

**Figure 3:**
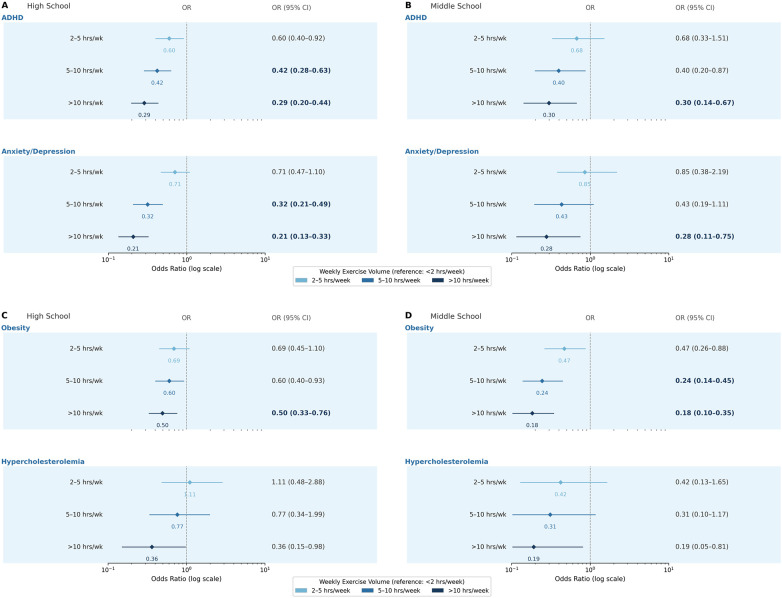

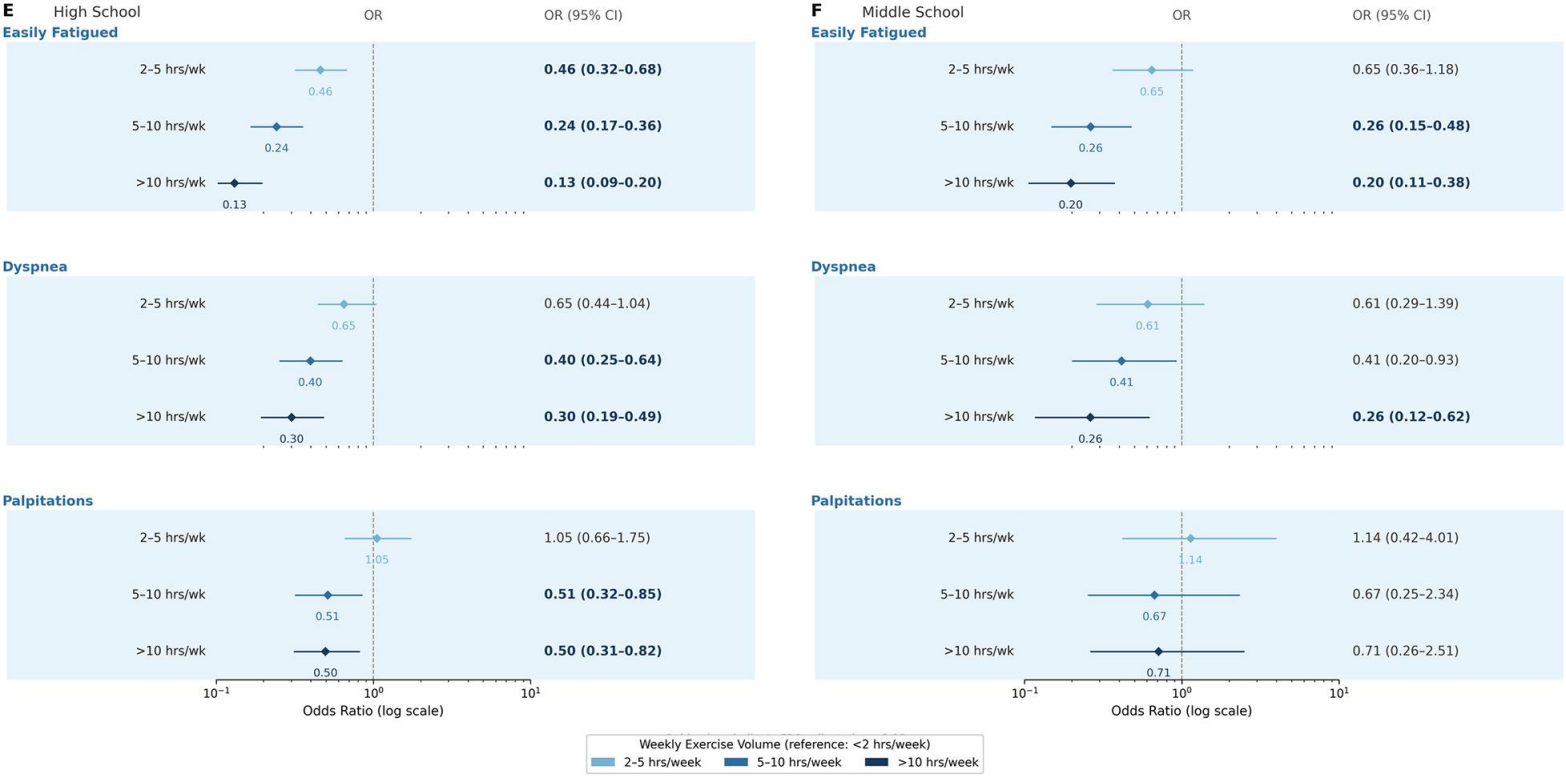
Association between weekly exercise volume and health outcomes in youth athletes. Forest plots show adjusted odds ratios (OR) with 95% confidence intervals from multivariable logistic regression, adjusted for age, sex, race, BMI, and area-level socioeconomic status (Distressed Communities Index). Reference group: <2 h/week. (A/B) Psychiatric comorbidities. (C/D) Cardiovascular risk factors. (E/F) Exertional symptoms. High school athletes shown in left panels **(A,C,E)**; middle school athletes shown in right panels **(B,D,F)**. Bold values indicate statistical significance at *α*<0.05.

Results from multivariable logistic regression analysis using athletes who exercised less than 2 h per week as the reference group and adjusting for age, race, sex, BMI, and SES proxied by DCI are shown in Tables 1, 2. The key findings are shown in Figure 3.

### Comorbidities logistic regression findings

Compared to the reference group, athletes exercising 5–10 h per week had lower odds of ADHD in high school (OR =  0.421, 95% CI: 0.284–0.634, *p* = 1.60 × 10^−4^). Those exercising more than 10 h per week showed reduced odds of ADHD in both middle school (OR = 0.298, 95% CI: 0.141–0.674, *p* = 0.010) and high school (OR = 0.288, 95% CI: 0.195–0.435, *p* = 2.19 × 10^−8^).

Greater exercise volume was associated with lower odds of anxiety or depression, with significant associations observed among those exercising 5–10 h per week in high school (OR = 0.318, 95% CI: 0.207–0.495, *p* = 3.07 × 10^−6^), and among those exercising more than 10 h per week in both middle school (OR = 0.277, 95% CI: 0.114–0.749, *p* = 0.029) and high school (OR = 0.208, 95% CI: 0.134–0.326, *p* = 7.89 × 10^−11^).

Athletes exercising 5–10 h per week had significantly lower odds of obesity in middle school compared to the reference group (OR = 0.245, 95% CI: 0.138–0.449, *p* = 2.15 × 10^−5^). An inverse association with obesity was also observed among those exercising more than 10 h per week in both middle school (OR = 0.183, 95% CI: 0.099–0.347, *p* = 1.57 × 10^−6^) and high school (OR = 0.496, 95% CI: 0.331–0.763, *p* = 5.42 × 10^−3^).

Although no associations were found statistically significant after FDR adjustment, those exercising more than 10 h per week exhibited a trend toward lower odds of hypercholesterolemia in both middle school (OR = 0.191, 95% CI: 0.050–0.810, *p* = 0.056) and high school (OR = 0.364, 95% CI: 0.150–0.983, *p* = 0.084).

### Exertional symptoms logistic regression

Reduced odds of exertional dyspnea were observed in high school athletes exercising 5–10 h per week (OR = 0.397, 95% CI: 0.252–0.636, *p* = 5.06 × 10^−4^), and in both middle and high school athletes exercising more than 10 h per week (OR = 0.262, 95% CI: 0.116–0.623, *p* = 8.15 × 10^−3^ and OR = 0.302, 95% CI: 0.192–0.486, *p* = 4.49 × 10^−6^, respectively). High school athletes exercising 2–5 h per week had significantly lower odds of feeling easily fatigued (OR = 0.461, 95% CI: 0.316–0.679, *p* = 4.37 × 10^−4^). Athletes exercising 5–10 h per week had significantly lower odds of feeling easily fatigued in both middle school (OR = 0.263, 95% CI: 0.148–0.480, *p* = 6.26 × 10^−5^) and high school (OR = 0.242, 95% CI: 0.165–0.357, *p* = 1.94 × 10^−11^). Additionally, athletes exercising more than 10 h per week had even lower odds of feeling easily fatigued in both middle school (OR =  0.197, 95% CI: 0.105–0.375, *p* = 4.94 × 10^−6^) and high school (OR =  0.131, 95% CI: 0.087–0.197, *p* = 5.69 × 10^−21^).

Compared to the reference group, high school athletes exercising 5–10 h and more than 10 h per week had lower odds of exertional palpitations (OR = 0.512, 95% CI: 0.316–0.854, *p* = 0.032 and OR = 0.496, 95% CI: 0.311–0.819, *p* = 0.020, respectively).

### ECG and cardiovascular exam logistic regression findings

Data was missing for 19 ECG outcomes and 331 murmur outcomes in the middle school stratum. In the high school stratum, data was missing for 33 ECG outcomes and 852 murmur outcomes. The participants with missing data were excluded from the two strata, and logistic regression found that no associations remained statistically significant after FDR adjustment for multiple comparisons for ECG and cardiovascular exam outcomes. However, several nominal trends were observed. Among middle school athletes, those exercising more than 10 h per week exhibited a nominal trend toward lower odds of ECG T-wave inversion compared to the reference group (OR = 0.054, 95% CI: 0.002–0.643, *p* = 0.065) (Figure 3). Among high school athletes, those exercising more than 10 h per week showed a nominal trend toward higher odds of murmur detection on physical exam (OR = 3.843, 95% CI: 1.411–15.83, *p* = 0.065). Additionally, high school athletes exercising 5–10 h per week (OR = 0.175, 95% CI: 0.036–0.935, *p* = 0.075) and more than 10 h per week (OR = 0.180, 95% CI: 0.044–0.914, *p* = 0.065) showed a nominal trend toward lower odds of ECG early repolarization compared to the reference group.

### Sensitivity analysis

A sensitivity analysis testing the interaction between school level and exercise volume in a combined unstratified model yielded no statistically significant interaction terms after FDR-adjustment.

## Discussion

In this cross-sectional study of 7,048 youth athletes, increased weekly physical activity volume was associated with lower odds of obesity and psychiatric comorbidities in both middle and high school athletes. Athletes exercising more than 10 h per week also exhibited a nominal trend towards lower odds of hypercholesterolemia in both middle and high school groups. While not statistically significnat, these findings are consistent with previous studies on adults demonstrating that physical activity is associated with improved metabolic health and lower prevalence of obesity and hypercholesterolemia, both of which have long-term benefits in preventing future coronary artery disease (16, 17). Greater exercise volume has also been associated with a lower prevalence of ADHD, anxiety, and depression in prior studies (18, 19), which is consistent with the associations observed in this cohort of youth athletes. Notably, this cohort represents adolescents presenting for PPE screening rather than exclusively competitive athletes, and the reference group of less than 2 h exercise per week may include mainly sedentary individuals rather than low-level athletes.

Increased weekly exercise volume was also associated with lower odds of exertional symptoms, which is consistent with previous findings that higher cardiorespiratory fitness developed through regular physical activity is associated with improved exercise tolerance and fewer exertional symptoms in youth (20). Specifically, this study found that both middle and high school athletes who exercised more than 10 h per week had lower odds of exertional dyspnea and feeling easily tired compared to the reference group, suggesting that these associations are present early in adolescence and persist through high school. These findings are consistent with evidence that physical deconditioning is associated with greater exertional dyspnea burden in adolescents (21), and lower activity levels may similarly contribute to the higher prevalence of symptoms seen in less active individuals in this youth athlete cohort. Further associations with lower odds of exertional palpitations were observed in high school athletes. The mechanisms underlying these associations are unclear, and direct evidence linking exercise volume to reducing palpitations specifically in youth athletes is limited. These findings should be interpreted as hypothesis-generating and may reflect the greater physiological and cardiovascular conditioning that develops with sustained athletic training in older adolescents, though this remains speculative in the absence of objective fitness measures.

Several associations between exercise volume and cardiovascular risk factors differed between middle and high school athletes, reflecting the physiological changes that occur across adolescent development. Cardiovascular adaptations to exercise are less pronounced in younger peripubertal athletes, with heart rate-mediated cardiac output increases being the dominant mechanism in this age group, while more advanced structural and functional cardiac adaptations develop progressively with age and continued training (22). The directionally consistent nominal trend toward lower odds of hypercholesterolemia observed across both school groups, alongside the stronger and more numerous significant cardiovascular and exertional symptom associations, in high school athletes, may reflect the cumulative associations between exercise volume and cardiometabolic health that develops with physiological maturation. These developmental differences emphasize the importance of age-stratification when examining exercise-related outcomes in youth populations.

Among middle school athletes, those who exercised more than 10 h per week showed a nominal trend toward lower odds of ECG T-wave inversion that did not reach statistical significance after FDR correction. Notably, T-wave inversion carries age-dependent clinical significance according to the International Criteria. While T-wave inversion in leads V1-V3 is considered a normal variant in athletes ≤16 years of age, it carries greater clinical significance in other leads or older adolescents, a distinction that should be considered when interpreting this finding across middle and high school groups (23, 24). The recently published 2026 EAPC/AEPC consensus statement on cardiac evaluation of pediatric athletes further classifies normal (V1–V3), borderline (V1–V4, age dependent), and abnormal (other leads excluding V1, III, and aVR) T-wave inversion findings in young athletes (25). As lead-specific ECG data were not available in this dataset, further classification of T-wave inversion findings according to this framework was not possible.

Among high school athletes, nominal trends toward lower odds of ECG early repolarization were observed among those exercising more than 5 h per week, though these associations did not reach statistical significance after FDR correction. Based on the international criteria, ECG early repolarization findings in trained athletes are considered to be physiologic (23, 24). Conversely, the presence of these ECG early repolarization findings in those who exercise fewer hours may reflect metabolic or deconditioning-related influences rather than athletic adaptation. These ECG adaptations are consistent with well-described cardiac remodeling in response to sustained exercise. Regular endurance exercise has been found to increase venous return and preload, potentially leading to volume overload of the ventricles and compensatory cardiac remodeling (26). Simultaneously, peripheral vascular resistance may decrease with blood volume expansion, which may reduce afterload and further promotes structural and electrical cardiac adaptations (27). These hemodynamic changes may provide a physiological basis for the ECG findings observed in athletes in this study, though cardiac adaptation also depends on sport type and years of training history which were not available in this dataset.

Nominal trends toward higher odds of murmur detection were observed in high school athletes exercising more than 10 h per week, though these associations did not reach statistical significance after FDR correction. This trend should be interpreted cautiously given several methodological considerations. Murmurs were recorded as binary findings, limiting the ability to distinguish physiologic from pathologic causes. This finding may reflect physiologic flow murmurs related to increased stroke volume and cardiac output in more conditioned athletes, which may be more readily detected on auscultation (26). Alternatively, this association may reflect detection bias, as more competitive athletes may receive more thorough cardiovascular evaluations, or inter-observer variability in auscultation technique across multiple PPE screening sites. The absence of this association in middle school athletes may suggest that hemodynamic adaptations driving flow murmurs require a longer duration of athletic training before they are detectable on auscultation. While most murmurs in youth are innocent, a workup is necessary to rule out potentially fatal pathology, with the American Heart Association guidelines recommending annual athletic prescreening for various risk factors and use of 12-lead-ECG or echocardiography if initial screening is concerning for possible underlying CVD (28–30). Providers should be aware of the importance of rescreening youth athletes to help mitigate the instances where youth athletes may have screened negative for any risk factors for SCD, but develop cardiovascular pathology later in adolescence or young adulthood.

### Limitations

There are several limitations to this study. First, the HeartBytes dataset is limited by the demographic homogeneity of its participants. Since the majority of individuals screened were White and male, this limits the generalizability of the results, and sex-specific effects were not formally evaluated. Many comorbidities and exertional symptoms in this database were recorded as a “Yes/No” based on patient and guardian recall from previous medical encounters. This may introduce recall bias and limit reproducibility given the inability to verify diagnostic criteria of comorbidities across patients. Additionally, the self-reported nature of physical activity and symptoms introduces the potential for response bias. Athletes who exercise less regularly may be less likely to recognize or report symptoms, and the fixed survey format may not have captured the full range of exercise-related symptoms. ECG findings also did not have lead-specific distributions, precluding stratification of T-wave inversion by lead location. This is particularly relevant given that the 2017 International Criteria specifies physiologic T-wave inversion as limited to leads V1–V3 in athletes ≤16 years of age, and the recently published 2026 EAPC/AEPC consensus statement on pediatric athletes further proposes a nuanced age-specific and lead-specific classification framework for T-wave inversion that could not be applied to this dataset (23, 25). Additionally, lead-specific T-wave inversion data were not available to assess whether findings differed by sex, which represents an important limitation given emerging evidence on sex-specific ECG patterns in female athletes (31, 32). The physical activity measure captured only total weekly hours and did not account for exercise intensity, sport type, training vs. recreational activity, or competition level, all of which may influence cardiovascular adaptation. Since the exercise volume categories used in this study were pre-defined by the HeartBytes screening questionnaire, they do not directly align with established frameworks such as WHO physical activity guidelines or the ESC sports cardiology classification of recreational, competitive, and elite athletes (33, 34). This limits direct comparability with studies using standardized exercise volume classifications. Future studies should consider incorporating these guideline frameworks, and also measuring these exercise dimensions separately to better characterize the dose-response relationship between athletic training and cardiovascular health outcomes. Given the exploratory nature of this cross-sectional analysis, the number of outcomes examined, and the self-reported nature of comorbidity ascertainment, FDR-adjusted *p*-values were used as the primary basis for inference and findings should be interpreted in this context. Lastly, since participants were self-selected volunteers, the results may overrepresent athletes who have increased awareness of cardiovascular risk factors, which limits the generalizability of these findings to the broader youth athlete population.

## Conclusion

In this study of the association of physical activity volume and cardiovascular risk factors in youth athletes, greater weekly exercise volume was associated with lower odds of metabolic comorbidities, psychiatric conditions, and exertional symptoms. Notably, several of these associations differed between middle and high school athletes, highlighting the importance of age-stratified interpretation of cardiovascular risk factors and symptoms in the adolescent population. While nominal trends toward higher odds of murmur detection were observed in high school athletes exercising more than 10 h per week, this finding did not reach statistical significance and warrants further investigation to clarify the clinical significance of these findings. Additional research is needed to better understand the clinical trajectory of exercise-associated cardiac findings in youth athletes and to inform evidence-based pre-participation screening guidelines for this population.

## Data Availability

The data analyzed in this study is subject to the following licenses/restrictions: The dataset analyzed during the current study is available from the corresponding author upon reasonable request. The database can be found at https://simonsheart2.heartbytes.org/login. Requests to access these datasets should be directed to info@simonsheart.org.

## References

[1] Vos T Lim SS Abbafati C Abbas KM Abbasi M Abbasifard M. Global burden of 369 diseases and injuries in 204 countries and territories, 1990–2019: a systematic analysis for the global burden of disease study 2019. Lancet. (2020) 396:1204–22. 10.1016/S0140-6736(20)30925-9 33069326 PMC7567026

[2] Arshad MM Ramphul K Dachepally R Almasri M Memon RA Sakthivel H. Five-year trends in risk factors for cardiovascular disease among adolescents in the United States. Arch Med Sci Atheroscler Dis. (2024) 9:e56–9. 10.5114/amsad/185775 38846057 PMC11155462

[3] Nystoriak MA Bhatnagar A. Cardiovascular effects and benefits of exercise. Front Cardiovasc Med. (2018) 5:135. 10.3389/fcvm.2018.00135 30324108 PMC6172294

[4] Martinez MW Kim JH Shah AB Phelan D Emery MS Wasfy MM. Exercise-Induced cardiovascular adaptations and approach to exercise and cardiovascular disease: jACC state-of-the-art review. J Am Coll Cardiol. (2021) 78(14):1453–70. 10.1016/j.jacc.2021.08.003 34593128

[5] Rodriguez-López AM Javier G Carmen P Esteban P Luisa G-C Tomas F. Athlete heart in children and young athletes. Echocardiographic findings in 331 cases. Pediatr Cardiol. (2022) 43:407–12. 10.1007/s00246-021-02736-5 34586455

[6] Castanheira J Valente-Dos-Santos J Costa D Martinho D Fernandes J Duarte J. Cardiac remodeling indicators in adolescent athletes. Rev Assoc Med Bras. (2017) 63:427–34. 10.1590/1806-9282.63.05.427 28724040

[7] McClean G Riding NR Ardern CL Farooq A Pieles GE Watt V. Electrical and structural adaptations of the paediatric athlete’s heart: a systematic review with meta-analysis. Br J Sports Med. (2018) 52:230. 10.1136/bjsports-2016-097052 28363973

[8] Sharma S Maron BJ Whyte G Firoozi S Elliott PM McKenna WJ. Physiologic limits of left ventricular hypertrophy in elite junior athletes: relevance to differential diagnosis of athlete’s heart and hypertrophic cardiomyopathy. J Am Coll Cardiol. (2002) 40:1431–6. 10.1016/S0735-1097(02)02270-2 12392833

[9] Makan J Sharma S Firoozi S Whyte G Jackson PG McKenna WJ. Physiological upper limits of ventricular cavity size in highly trained adolescent athletes. Heart. (2005) 91:495–9. 10.1136/hrt.2004.035121 15772210 PMC1768829

[10] Rowland T. Morphologic features of the “athlete’s heart” in children: a contemporary review. Pediatr Exerc Sci. (2016) 28:345–52. 10.1123/pes.2015-0239 26694944

[11] Barczuk-Falęcka M Małek ŁA Krysztofiak H Roik D Brzewski M. Cardiac magnetic resonance assessment of the structural and functional cardiac adaptations to soccer training in school-aged male children. Pediatr Cardiol. (2018) 39:948–54. 10.1007/s00246-018-1844-5 29520462 PMC5958145

[12] Merghani A Malhotra A Sharma S. The U-shaped relationship between exercise and cardiac morbidity. Trends Cardiovasc Med. (2016) 26:232–40. 10.1016/j.tcm.2015.06.005 26187713

[13] Drezner JA Ackerman MJ Anderson J. Electrocardiographic interpretation in athletes: the “Seattle criteria. Br J Sports Med. (2013) 47:122–4. 10.1136/bjsports-2012-092067 23303758

[14] Francis R. Data reveals rising economic “distress” across America despite post-pandemic growth. *Economic Innovation Group* (2023) Available online at: https://eig.org/dci-hub/ (Accessed June 9, 2026).

[15] Forså MI Bjerring AW Haugaa KH Smedsrud MK Sarvari SI Landgraff HW. Young athlete’s growing heart: sex differences in cardiac adaptation to exercise training during adolescence. Open Heart. (2023) 10:e002155. 10.1136/openhrt-2022-002155 36596623 PMC9814996

[16] Mann S Beedie C Jimenez A. Differential effects of aerobic exercise, resistance training and combined exercise modalities on cholesterol and the lipid profile: review, synthesis and recommendations. Sports Med. (2014) 44:211–21. 10.1007/s40279-013-0110-5 24174305 PMC3906547

[17] Niemiro GM Rewane A Algotar AM. Exercise and Fitness Effect on Obesity. Treasure Island, FL: StatPearls Publishing (2023). 30969715

[18] Mehren A Reichert M Coghill D Müller HHO Braun N Philipsen A. Physical exercise in attention deficit hyperactivity disorder—evidence and implications for the treatment of borderline personality disorder. Borderline Personal Disord Emot Dysregul. (2020) 7:1. 10.1186/s40479-019-0115-2 31921425 PMC6945516

[19] Schuch FB Vancampfort D. Physical activity, exercise, and mental disorders: it is time to move on. Trends Psychiatry Psychother. (2021) 43:177–84. 10.47626/2237-6089-2021-0237 33890431 PMC8638711

[20] Raghuveer G Hartz J Lubans DR Takken T Wiltz JL Mietus-Snyder M. Cardiorespiratory fitness in youth: an important marker of health: a scientific statement from the American Heart Association. Circulation. (2020) 142:e101–18. 10.1161/CIR.0000000000000866 32686505 PMC7524041

[21] Hengeveld VS van der Kamp MR Thio BJ Brannan JD. The need for testing-the exercise challenge test to disentangle causes of childhood exertional dyspnea. Front Pediatr. (2021) 9:773794. 10.3389/fped.2021.773794 35071131 PMC8770982

[22] Pieles GE Stuart AG. The adolescent athlete’s heart; A miniature adult or grown-up child? Clin Cardiol. (2020) 43:852–62. 10.1002/clc.23417 32643161 PMC7403711

[23] Sharma S Drezner JA Baggish A Papadakis M Wilson MG Prutkin JM. International recommendations for electrocardiographic interpretation in athletes. J Am Coll Cardiol. (2017) 69:1057–75. 10.1016/j.jacc.2017.01.015 28231933

[24] Basu J Malhotra A. Interpreting the athlete’s ECG: current state and future perspectives. Curr Treat Options Cardiovasc Med. (2018) 20:104. 10.1007/s11936-018-0693-0 30456469 PMC6244896

[25] Pieles GE Cavarretta E Orchard JJ Abela M Arbelo E Budts W. Cardiac evaluation of paediatric athletes. Eur Heart J. (2026) 47:2561–83. 10.1093/eurheartj/ehag188 41967042 PMC13225883

[26] Baggish AL Wood MJ. Athlete’s heart and cardiovascular care of the athlete: scientific and clinical update: scientific and clinical update. Circulation. (2011) 123:2723–35. 10.1161/CIRCULATIONAHA.110.981571 21670241

[27] Hellsten Y Nyberg M. Cardiovascular adaptations to exercise training. Compr Physiol. (2016) 6:1–32. 10.1002/j.2040-4603.2016.tb00672.x 26756625

[28] Frank JE Jacobe KM. Evaluation and management of heart murmurs in children. Am Fam Physician. (2011) 84:793–800. 22010618

[29] Maron BJ Thompson PD Ackerman MJ Balady G Berger S Cohen D. Recommendations and considerations related to preparticipation screening for cardiovascular abnormalities in competitive athletes: 2007 update: a scientific statement from the American Heart Association council on nutrition, physical activity, and metabolism: endorsed by the American College of Cardiology foundation. Circulation. (2007) 115:1643–55. 10.1161/CIRCULATIONAHA.107.181423 17353433

[30] Maron BJ Friedman RA Kligfield P Levine BD Viskin S Chaitman BR. Assessment of the 12-lead electrocardiogram as a screening test for detection of cardiovascular disease in healthy general populations of young people (12–25 years of age): a scientific statement from the American Heart Association and the American College of Cardiology. J Am Coll Cardiol. (2014) 64:1479–514. 10.1016/j.jacc.2014.05.006 25234655

[31] Davis AJ van der Linden R Mukhtar S Sardelich D Ungaro S Zorzi A. Sex differences in electrocardiograms of trained athletes: a systematic review and meta-analysis. Eur J Prev Cardiol. (2026) 33:1348–62. 10.1093/eurjpc/zwag004 41557583

[32] Orchard JJ Drezner JA Raju H Puranik R Gray B Brosnan M. Isolated anterior T-wave inversion in elite athletes: prevalence and clinical relevance by sex and sporting discipline. J Am Heart Assoc. (2025) 14:e042435. 10.1161/JAHA.125.042435 41025448 PMC12684483

[33] Chaput J-P Willumsen J Bull F Chou R Ekelund U Firth J. 2020 WHO guidelines on physical activity and sedentary behaviour for children and adolescents aged 5–17 years: summary of the evidence. Int J Behav Nutr Phys Act. (2020) 17:141. 10.1186/s12966-020-01037-z 33239009 PMC7691077

[34] Pelliccia A Sharma S Gati S Bäck M Börjesson M Caselli S. 2020 ESC guidelines on sports cardiology and exercise in patients with cardiovascular disease. Eur Heart J. (2021) 42:17–96. 10.1093/eurheartj/ehaa605 32860412

